# ERK is a negative feedback regulator for IFN-γ/STAT1 signaling by promoting STAT1 ubiquitination

**DOI:** 10.1186/s12885-018-4539-7

**Published:** 2018-05-31

**Authors:** Ying Zhang, Yelong Chen, Zhaoyong Liu, Raymond Lai

**Affiliations:** 1 Department of Pathology, Shantou University Medical College, 22 Xinling Road, Shantou, Guangdong Province China; 2 Department of Orthopaedics, First Affiliated Hospital of Shantou University Medical College, No.57 Changping Road, Shantou, 515041 Guangdong China; 3 Department of Pathology, University of Alberta, Edmonton, AB Canada

**Keywords:** Esophageal squamous cell carcinoma, STAT1, ERK, Proteasomal degradation, Prognostic

## Abstract

**Background:**

We recently reported that STAT1 plays a tumor suppressor role, and ERK was inversely correlation with STAT1 expression in esophageal squamous cell carcinoma (ESCC). Here, we investigated the mechanism(s) that are responsible for the ERK regulates STAT1 in ESCC.

**Methods:**

We performed the immunoprecipitation (IP) to detect the ubiquitin of STAT1 upon MEK transfection or U0126 treatment and co-IP to confirm the binding of STAT1 and ERK in ESCC cell lines.

**Results:**

We found evidence that the ubiquitin-proteasome pathway can efficiently degrade STAT1 in ESCC cells, as MG132 treatment rapidly and dramatically increased STAT1 expression in these cells. This process is not dependent on the phosphorylation of the two important STAT1 residues, Y701 and S727, as site-directed mutagenesis of these two sites did not affect STAT1 degradation. We also found that ERK promotes proteasome degradation of STAT1, supported by the observations that pharmacologic inhibition of ERK resulted in a substantial increase of STAT1 whereas expression of constitutively active ERK further reduced the STAT1 protein level. In addition to suppressing STAT1 expression, ERK limited STAT1 signaling by decreasing the production of IFNγ.

**Conclusion:**

To conclude, ERK is an effective negative regulator of STAT1 signaling in ESCC, by promoting its proteasome degradation and decreasing IFNγ production. Our data further supports that targeting ERK and/or STAT1 may be useful for treating ESCC.

**Electronic supplementary material:**

The online version of this article (10.1186/s12885-018-4539-7) contains supplementary material, which is available to authorized users.

## Background

Esophageal squamous cell carcinoma (ESCC) is one of the leading cancer worldwide, especially China [1]. Chaoshan area is the highest age-standardized incidence area in China [2]. The growth of ESCC is regulated via a complex network of signal transduction pathways.

The JAK-STAT (Signal transducers and activators of transcription) signaling pathway has been identified as a potential target for novel cancer therapies. STAT1 is a critical mediator of cytokine signaling [3]. STAT1 is a key mediator in gamma-interferon (IFNγ) signaling, which activates STAT1 by its phosphorylation. STAT1 has been reported as a tumor suppressor in breast cancer, multiple myeloma and leukemia [4]. We previously reported that STAT1 can promote ESCC cell apoptosis and inhibit cell growth and the STAT1^low^ expression patients in ESCC significantly correlates with a worse clinical outcome [5, 6]. Extracellular regulated protein kinases (ERK), a key regulator of mitogen-activated protein kinase (MAPK) signaling pathway, functions as an important anti-cancer therapeutic target in multiple malignant tumors, such as breast cancer, esophageal cancer [7]. In our previous research, we found that ERK can negative regulate STAT1 in ESCC and expression of ERK/p-ERK is adversely correlated with STAT1 expression. The ESCC patients with ERK ^low^/STAT1 ^high^ had the longest survival compared with other patients [7].

The function of the ubiquitin-proteasome pathway (UPP) involves removing damaged and redundant proteins from the cells. This process occurs in the regulation of several cellular processes, including cell proliferation, cell death, cell division, DNA repair, and cell differentiation [8]. Aberrations of this pathway occur in the pathogenesis of various human diseases, especially chronic inflammatory diseases and neurodegenerative disorders, even cancer [9]. Proteasome inhibitors have emerged as a novel chemotherapeutic agent for treating cancers. The peptide-aldehyde proteasome inhibitor MG132 (carbobenzoxyl-L-leucyl-L-leucyl-L-leucine), inhibiting 20S proteasome activity and effectively blocking the proteolytic activity of the 26S proteasome complex, has been reported to induce tumor cell apoptosis and thus it plays a crucial role in anti-tumor treatment [10].

In this study, the main purpose is to delineate the mechanisms by which IFN/STAT1 signaling is down-regulated by ERK in ESCC. In this study, we identified that ERK is a key regulator of STAT1, by means of promoting its proteasomal degradation and decreasing the production of IFNγ.

## Methods

### Cell lines

Human ESCC cell lines EC1 and KYSE150, were used in this study. EC1 cell lines was purchased from Hongkong University in 2011 and KYSE150 was purchased from DSMZ in 2012. They were maintained in RPMI 1640 or Dulbecco’s modified Eagle’s medium (DMEM) supplemented with 10% FBS (Invitrogen, Carlsbad, CA, USA). All cells were verified to be free of mycoplasma contamination.

### siRNA, plasmid constructs and drugs

STAT1 siRNA and scramble RNA were purchased from Santa Cruz Biotechnology (Santa Cruz, CA, USA). Transfection of siRNA was performed using Lipofectamine RNAimax (Invitrogen, Carlsbad, CA, USA) according to the manufacturer’s instructions. Plasmids including eGFP-STAT1, eGFP-STAT1Y701F, eGFP-STAT1S727A, Flag-STAT1β were purchased from Addgene (Cambridge, MA, USA). Flag-tagged, constitutive-active STAT1 (or STAT1C) cloned into the backbone of pcDNA3.1 was a gift from Dr. Toru Ouchi (Roswell Park Cancer Institute, University at Buffalo, NY, USA). The constitutively active MEK-1 (HA-ca-MEK) vector was a gift from Dr. Nathalie Rivard (Université de Sherbrooke, Québec, CA). The proteasome inhibitor N-carbobenzoxyl-L-leucinyl-L-norleucinal (MG132) was purchased from Calbiochem (La Jolla, CA, USA) and 5-Aza-2′-deoxycytidine (5-Aza) was purchased from Sigma (St Louis, MO, USA). U0126, a Mitogen-activated protein kinase 1 (MEK1) inhibitor was purchased from Sigma (St Louis, MO, USA).

### Co-immunoprecipitation, immunoprecipitation and western blot analysis

Co-immunoprecipitation, immunoprecipitation and Western blot analysis were performed as described previously [5]. Antibodies reactive with human β-actin, caspase-9, caspase-3, Bcl-2, Bcl-xL, STAT1, phospho-STAT1 serine 727 (or p-STAT1 S727), phospho-STAT1 tyrosine 701 (or p-STAT1 Y701), phospho-ERK (or p-ERK), ERK, Ubiquitin (or Ub), phospho-JAK2 (or p-JAK2), JAK2, Human influenza hemagglutinin (or HA) and Poly ADP ribose polymerase (PARP) were purchased from Cell Signaling.

### Cell growth and Colony formation assay

To evaluate the effect of MG132 on the growth of ESCC cell lines, cell viability was determined by using the 3-(4,5-dimethylthiazol-2-yl)-5-(3-carboxymethoxyphenyl)-2-(4-sulfophenyl)-2H-tetrazolium (MTS) assay (Promega, Madison, WI) and trypan blue assay (sigma, St Louis, MO, USA) according to the manufacturer’s protocol. Colony formation assay was performed as described previously [5].

### Quantitative RT-PCR

Using the RNeasy Mini Kit (Qiagen, Valencia, CA, USA), total cellular RNA was extracted from cells following the manufacturer’s protocol. Primer sequence for IFNγ is: Forward: 5′- TGACCAGAGCATCCAAAAGA-3′, Reverse: 5′-CTCTTCGACCTCGAAACAGC-3′. IFNγ receptor: Forward: 5′-TCTTTGGGTCAGAGTTAAAGCCA-3′, Reverse: 5′-TTCCATCTCGGCATACAGCAA-3′. Human GAPDH was used as control.

### Immunofluorescence and confocal microscopy

Immunofluorescence was performed as previously described [5]. Cells were visualized with a Zeiss LSM 710 confocal microscope at the Core Cell Imaging Facility, Cross Cancer Institute, Alberta, Canada.

### Flow cytometry

Apoptosis was measured by using the FITC-conjugated Annexin V/PI assay kit (Invitrogen, Carlsbad, CA, USA) and flow cytometry. After treatment with scramble RNA or siRNA STAT1 for 24 h, cells were treated with 10 μM MG132 for 24 h and then collected according to the manufacturer’s protocol.

### Enzyme-linked immunosorbent assay (ELISA)

The supernatant of cell suspension from harvested cells were collected, the level of IFNγ was analyzed by ELISA using the commercially available ELISA kits in a 96-well microplate (eBioscience, San Diego, CA, USA) at an optical density of 450 nm, according to the manufacturer’s protocol.

### Analysis of ESCC data in cBioportal for cancer genomics database

The cBioPortal for Cancer Genomics is an open-access downloaded bio-database, providing visualization and analyzing tool for large-scale cancer genomics data sets (www.cbioportal.org). This portal collected records that were derived from 147 individual cancer studies, in which 31 types of cancer were analyzed, which included over 21,000 samples. Analysis of the 185 esophageal cancer samples from this database was performed in silico.

### Statistical analysis

Data was expressed as mean ± standard deviation. The prognostic significance of the expression of various markers was analyzed using the Kaplan Meier’s analysis. Differences among the treatment groups were assessed using ANOVA and the appropriate statistical software (SPSS, IBM, USA). A *p*-value of ≤0.05 was considered as statistically significant.

## Results

### MG132 increases the protein expression of STAT1 in ESCC cell lines

To investigate the mechanism(s) by which STAT1 is down-regulated in ESCC, we questioned if the *STAT1* gene is silenced via gene methylation. Thus, we treated two ESCC cell lines EC1 and KYSE150 with 1–10 μM 5-Aza for 0–48 h. By Western blots and quantitative RT-PCR, we did not find any appreciable change in STAT1 nor phospho(p)-STAT1 expression, suggesting that gene methylation does not play a role in suppressing STAT1 expression in ESCC (Additional file 1: Figure S1).

**Fig. 1:**
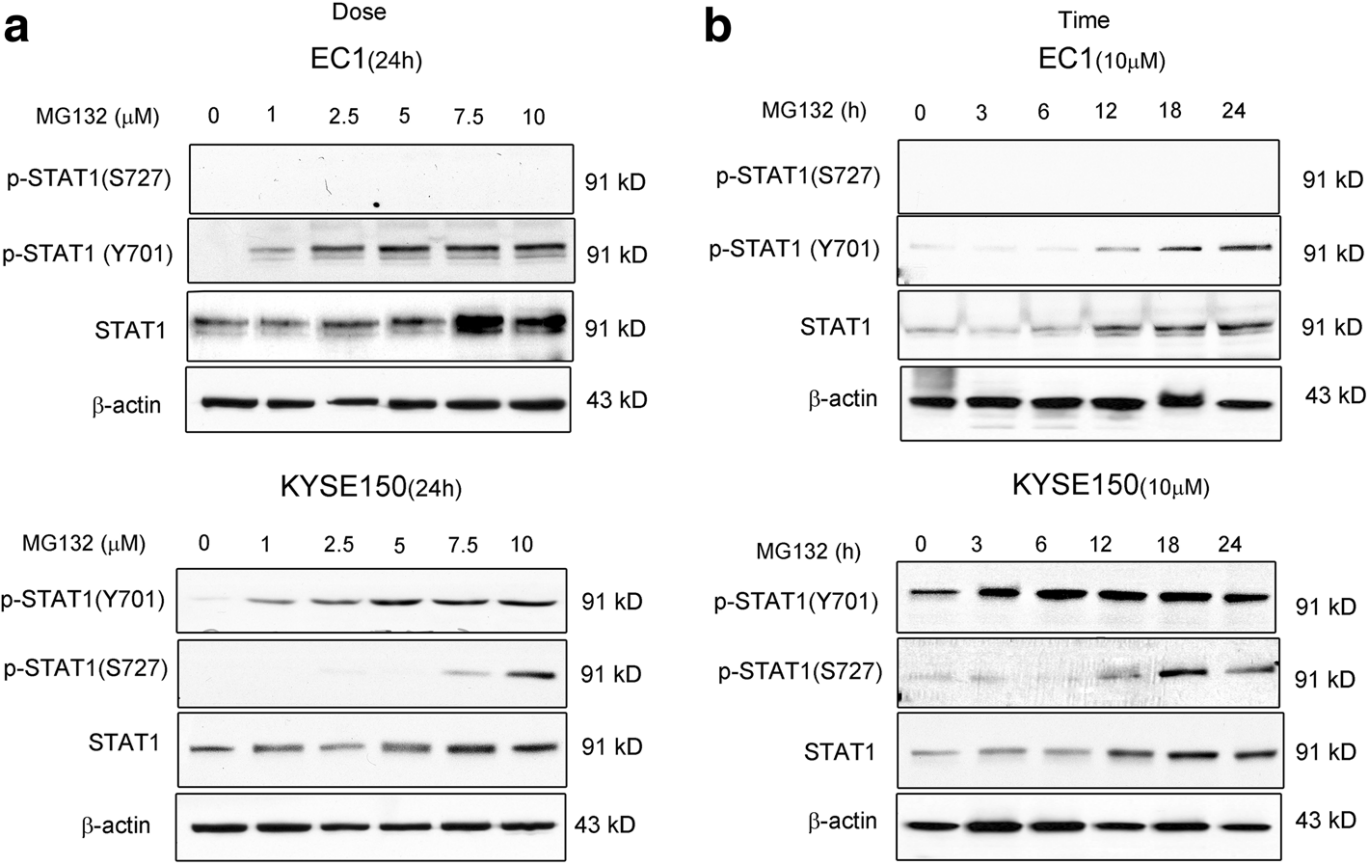
MG132 increases expression of p-STAT1 and STAT1 in ESCC cell lines. Western blot analysis demonstrates that the dose-dependent and time-dependent elevation of STAT1 induced by MG132. **a.** EC1 and KYSE150 cell lines were treated in the presence of 0–10 μM MG132 for 24 h. Total cell lysates were then prepared for immunoblot detection of p-STAT1^Y701^ and p-STAT1^S727^, STAT1 and β-actin. **b**. ESCC cell lines were treated with 10 μM MG132 and cells were harvested for immunoblot analysis at different time intervals. **c.** *STAT1* transfection into EC1 and KYSE150 cells resulted in a dramatic increase in the levels of p-STAT1 and STAT1. Similar results were observed in three independent experiments. (E.V.: Empty vector)

In view of one previous report that STAT1 can be degraded via the ubiquitin-proteosome pathway in mouse embryonic fibroblasts [11], we tested if this mechanism contributes to the low expression level of STAT1 in ESCC. Thus, we treated EC1 and KYSE150 with varying concentrations (1–10 μM) of MG132 for 24 h. By Western blots, we found that the STAT1 protein level in all cell lines was dramatically up-regulated in a dose-dependent manner, and this STAT1 up-regulation was detectable at an MG132 concentration as low as 1 μM (Fig. 1a). Furthermore, MG132 induced an increase in STAT1 in time-dependent manner (Fig. 1b). With the exception of EC1, a cell line that did not express p-STAT1^S727^, the STAT1 phosphorylation level at Y701 and S727 generally increased in parallel with the total protein level of STAT1. These findings suggest that there are mechanism(s) that constitutively activate STAT1 in ESCC cells at the steady state.

### ERK promotes polyubiquitination of STAT1 independent of STAT1 phosphorylation

**Fig. 2:**
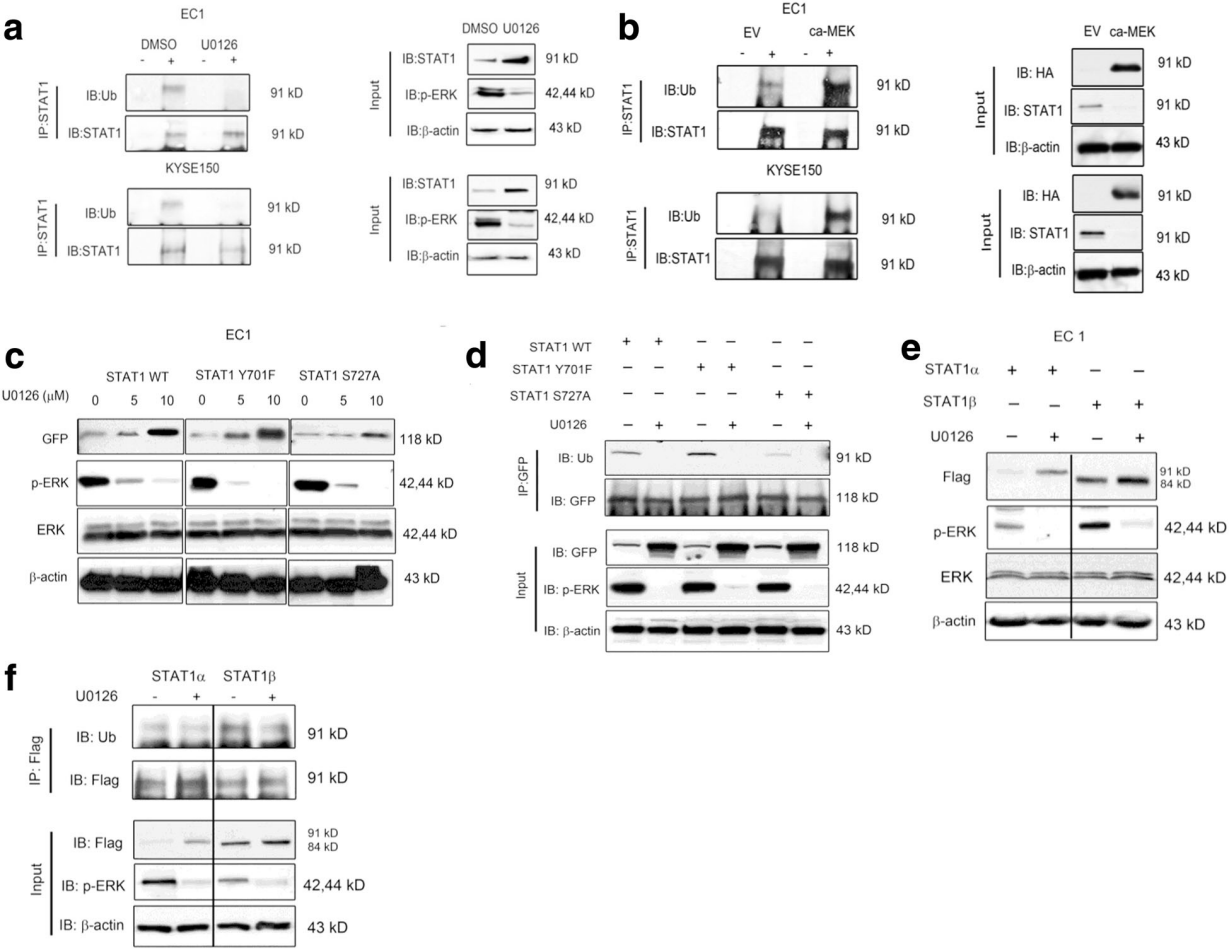
ERK promotes polyubiquitination of STAT1 independent of STAT1 phosphorylation. **a**. Immunoprecipitation experiments were performed to evaluate the level of ubiquitination of STAT1 in EC1 and KYSE150 cells treated with or without U0126 for 2 h. **b**. HA-ca-MEK plasmid was transfected into both ESCC cell lines together with empty vector (E.V.). STAT1 ubiquitination was detected by immunoprecipitation with anti-STAT1 antibody and immunoblotting with an anti-Ub antibody. **c**. GFP-STAT1 (WT), GFP-STAT1 (Y701F), or GFP-STAT1 (S727A) were transfected into EC1 cells together with increasing amounts of U0126. The protein level of GFP, p-ERK and ERK in the lysates was determined by Western blot. **d.** GFP-STAT1 (WT), GFP-STAT1 (Y701F), or GFP-STAT1 (S727A) were transfected into EC1 cells together with or without U0126. STAT1 ubiquitination was detected by immunoprecipitation with anti-GFP antibody and immunoblotting with anti-Ub antibody. **e**. Flag-STAT1α and Flag-STAT1β were transfected into EC1 together with or without U0126. The protein level of Flag, p-ERK and ERK in the lysates was determined by Western blot. **f**. Flag-STAT1α and Flag-STAT1β were transfected into EC1 with or without U0126. STAT1 ubiquitination was detected by immunoprecipitation with anti-Flag antibody and immunoblotting with anti-Ub antibody. Similar results were observed in three independent experiments

We performed immunoprecipitation and Western blots to detect STAT1 ubiquitination in EC1 and KYSE150 cells. As shown in Fig. 2a, STAT1 ubiquitination was decreased in the presence of U0126 compared to the negative controls. Moreover, transfection of the constitutively-activated MEK/ERK plasmid increased STAT1 polyubiquitination, compared with the empty vector **(**Fig. 2b**)**. Taken together, these results support the concept that ERK activation promotes polyubiquitination and proteasomal degradation of STAT1.

As mentioned, one previous study using mouse embryonic fibroblasts has shown that ERK phosphorylates STAT1 at S727 and targets it for proteasomal degradation [11]. To determine whether the two phosphorylation sites of STAT1 (Y701 and S727) contribute to STAT1 polyubiquitination, we transfected plasmids encoding GFP-tagged STAT1 or the two STAT1 mutants (S727A and Y701F) into the EC1 cells, and the results are illustrated in Fig. 2c. As expected, treatment of U0126 effectively decreased the amount of p-ERK, and this change correlated with a reciprocal increase in GFP and STAT1 proteins. The levels of both STAT1^S727A^ and STAT1^Y701F^ increased in response to the U0126 treatment. In addition, silencing of ERK by inhibitor U0126 decreased the polyubiquitination of STAT1, either in the form of wild-type and mutants (Fig. 2d). These findings suggest that the degradation of STAT1 mediated by ERK is independent of the phosphorylation at Y701 or S727. STAT1β is a C-terminal-truncated version of STAT1 that lacks S727, but retains Y701 [12] . To further support the concept that phosphorylation of STAT1^S727^ is not required for ERK-mediated STAT1 proteasomal degradation, we transfected Flag-tagged STAT1α and STAT1β plasmids into EC1 cells with or without U0126. As shown in Fig. 2e, we found that U0126 can increase both exogenous STAT1α and STAT1β in both cell lines; in addition, ERK can promote the polyubiquitination of both STAT1α and STAT1β **(**Fig. 2f**)**. These results further support that active ERK can mediate STAT1 degradation that is independent of the phosphorylation of S727.

### ERK binds to STAT1 in ESCC

**Fig. 3:**
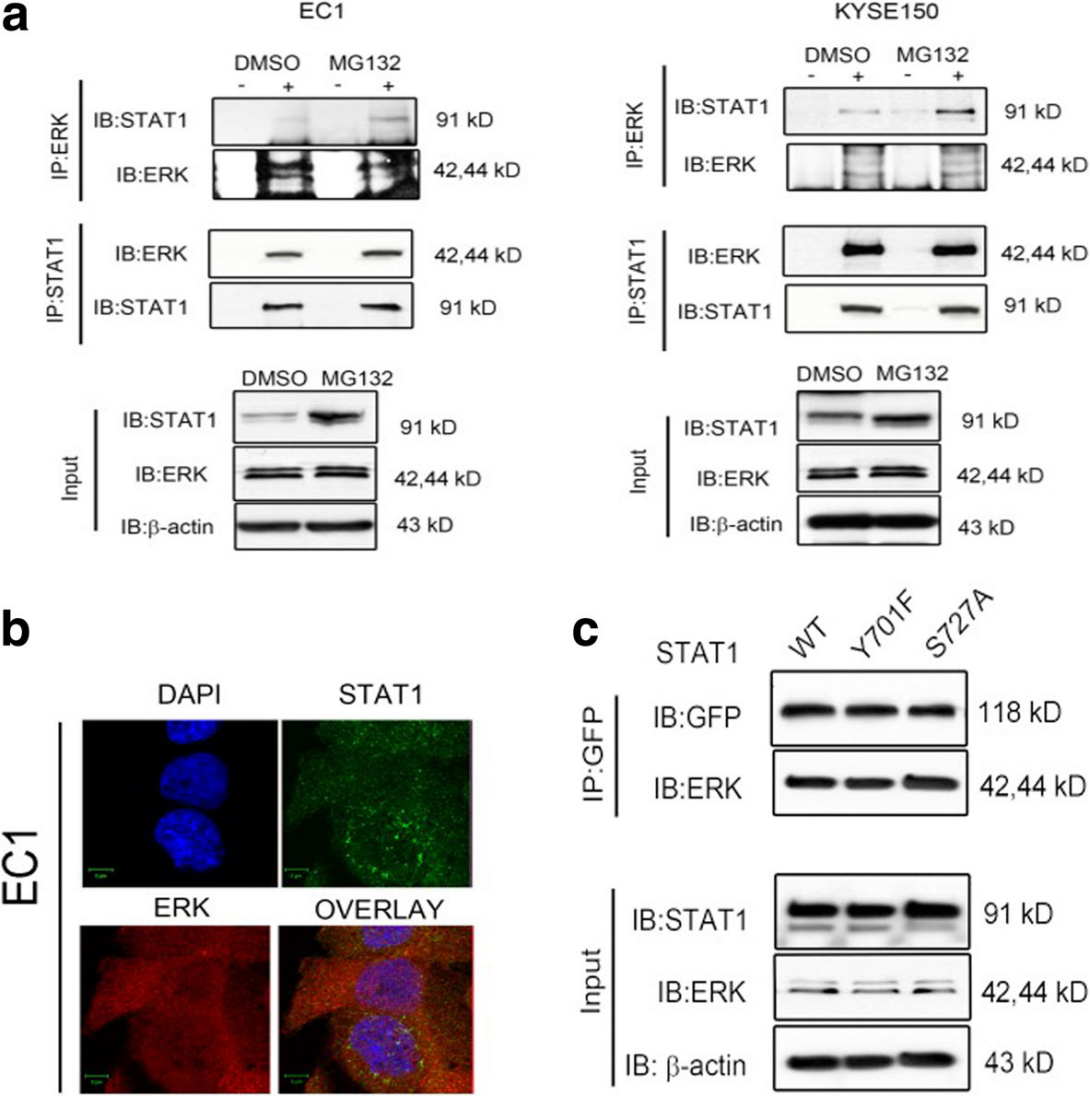
ERK binds to STAT1 in ESCC. **a.** The interaction of endogenous ERK and STAT1 was investigated by using co-immunoprecipitation experiments in EC1 and KYSE150 cells treated with or without MG132. Co-immunoprecipitation was carried out using negative control IgG, anti-STAT1, or anti-ERK antibody. **b**. Co-localization of ERK and STAT1 in EC1 cells were examined by using confocal microscopy (scale bar, 5 μm). **c.** GFP-STAT1 (WT), GFP-STAT1 (Y701F), or GFP-STAT1 (S727A) were transfected into EC1 cells. Co-immunoprecipitation was performed using GFP antibody and immunoblotting was done using an anti-ERK antibody. Similar results were observed in three independent experiments. (E.V.: Empty vector)

To further delineate the mechanisms underlying ERK-mediated STAT1 proteasomal degradation, we examined whether ERK binds to STAT1. Co-immunoprecipitation experiments were performed using EC1 and KYSE150 cells, with or without MG132 treatment. As shown in Fig. 3a, immunoprecipitation of ERK pulled down STAT1 in both cell lines, and MG132 treatment appreciably increased the amount of STAT1 bound to ERK, probably due to the fact that the total amount of STAT1 increased in response to proteasomal inhibition by MG132. Reciprocal pull-down experiments showed essentially the same results, except that we did not see a MG132-induced increase in the amount of ERK bound to STAT1, probably due to the fact that the protein level of ‘bioavailable’ STAT1 was substantially lower than that of ERK. Moreover, the co-localization of ERK and STAT1 was confirmed by confocal microscopy (Fig. 3b). To determine whether the phosphorylation status of STAT1 is required for the ERK-STAT1 interaction in ESCC cells, we transfected EC1 cells with wild-type STAT1, STAT1^Y701F^ or STAT1^S727A^. All of the plasmids used were GFP-tagged. Results are illustrated in Fig. 3c. ERK was pulled down along with STAT1 as well as STAT1 mutants. These results indicated that STAT1 can bind to ERK independent of the phosphorylation status of STAT1 at the Y701 or S727, two sites known to be important for the function of STAT1**.** These findings are in parallel with the previous experiments in which we showed that ERK can mediate STAT1 degradation independent of the two STAT1 phosphorylation sites mutants.

### ERK is a negative feedback regulator for IFN-γ/STAT1 signaling

**Fig. 4:**
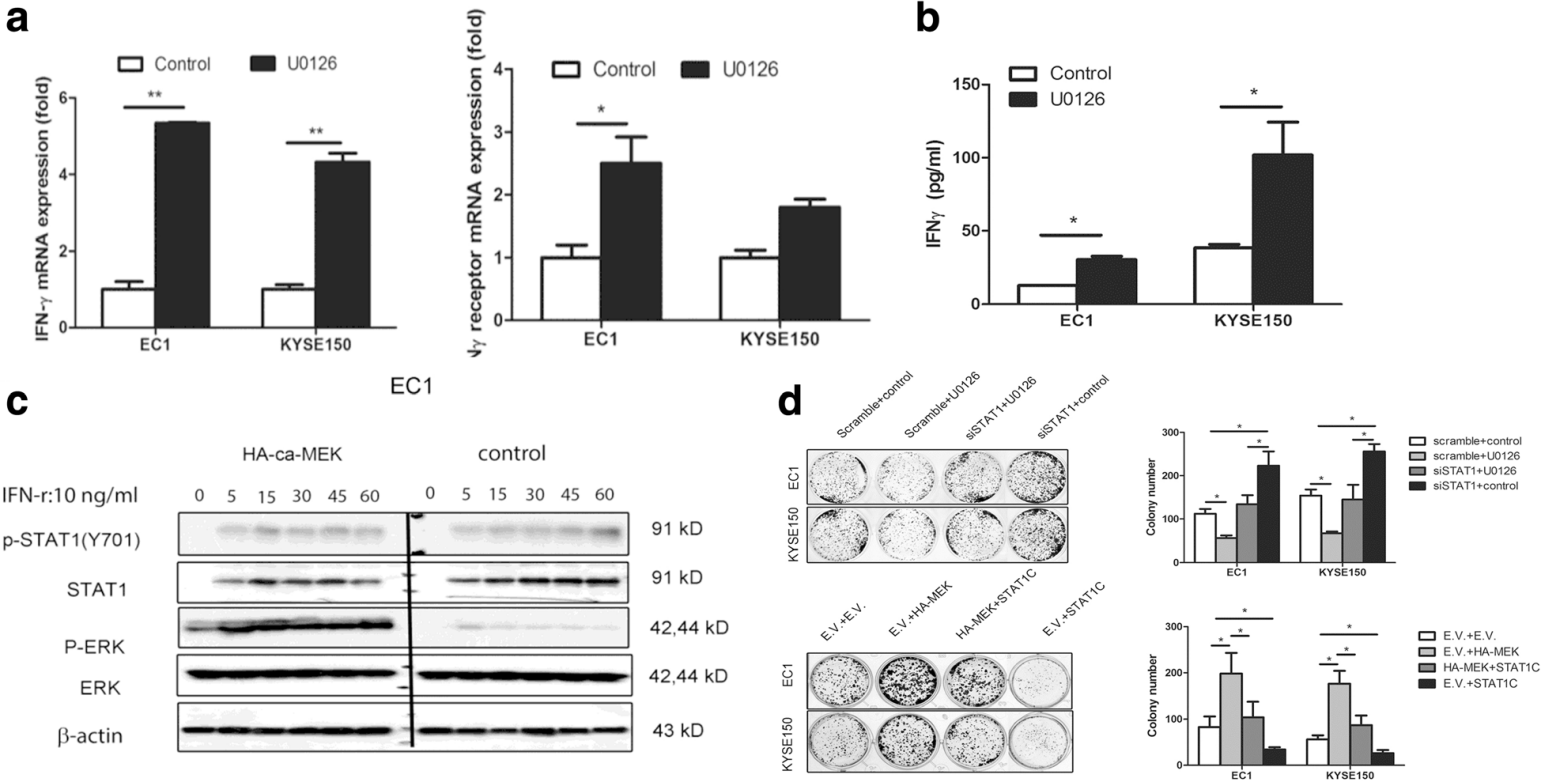
ERK is a negatively feedback regulator for IFN-γ/STAT1 signaling. **a.** ESCC cell lines EC1 and KYSE150 were treated with U0126 for 3 h, the IFNγ and the receptor mRNA expression was detected by qRT-PCR. **b**. The active form of IFNγ was detected by ELISA in both cell lines. **c**. ESCC cell lines EC1 transfected with HA-ca-MEK or empty vector were treated with IFN-γ (10 ng/ml) for 0–60 min. The expression of p-STAT1, STAT1 was measured by Western blot. **d**. The function study of ERK/STAT1 correlation is detected by colony formation assay. We treated the cells with STAT1 siRNA or scramble RNA and U0126 or control. Similarly, we transfected of STAT1C or empty vector and then treated the cells with constitutive active MEK (HA-MEK) or empty vector together. Then we cultured the cells for 10 days. Similar results were observed in three independent experiments. (*p < 0.05; **p < 0.01) (E.V.: Empty vector)

The ERK signaling pathway has been reported to play a crucial role in IFNγ/STAT1 signaling in human macrophages [13]. We hypothesized that, in addition to decreasing STAT1 expression, ERK may further down-regulate STAT1 signaling by suppressing IFNγ production. Our data is in support of this concept. Using quantitative RT-PCR, we found that inhibition of ERK by U0126 increased the expression of IFNγ and IFNγ receptor in both ESCC cell lines (Fig. 4a). As shown in Fig. 4b, similar results were found when we performed the ELISA to detect the active form of IFNγ. As shown in Fig. 4c, we also found evidence that IFNγ up-regulated p-ERK, which appears to serve as a gatekeeper to prevent over-stimulation of STAT1, which is known to provide potent pro-apoptotic signal in ESCC cells [9]. To demonstrate the effectiveness of ERK in blocking IFNγ-mediated activation of STAT1, we transfected EC1 with the constitutively active ERK vector before the addition of IFNγ to the cell culture. As shown in Fig. 4c, the expression of constitutively active ERK substantially dampened the up-regulation of p-STAT1 induced by IFNγ. Moreover, we performed the colony formation to evaluate the biological effect of ERK on STAT1 in both cell lines. As shown in Fig. 4d, U0126 significantly diminished the clonogenic ability of ESCC cell with STAT1 knockdown by siRNA; at the same time, constitutive-active MEK significantly increased the clonogenic ability of ESCC cells transfected with *STAT1C* (*p* < 0.05). Taken together, these findings suggest that ERK is a key regulator of STAT1 function in ESCC cells.

### MG132-induced apoptosis in ESCC is STAT1-dependent

**Fig. 5:**
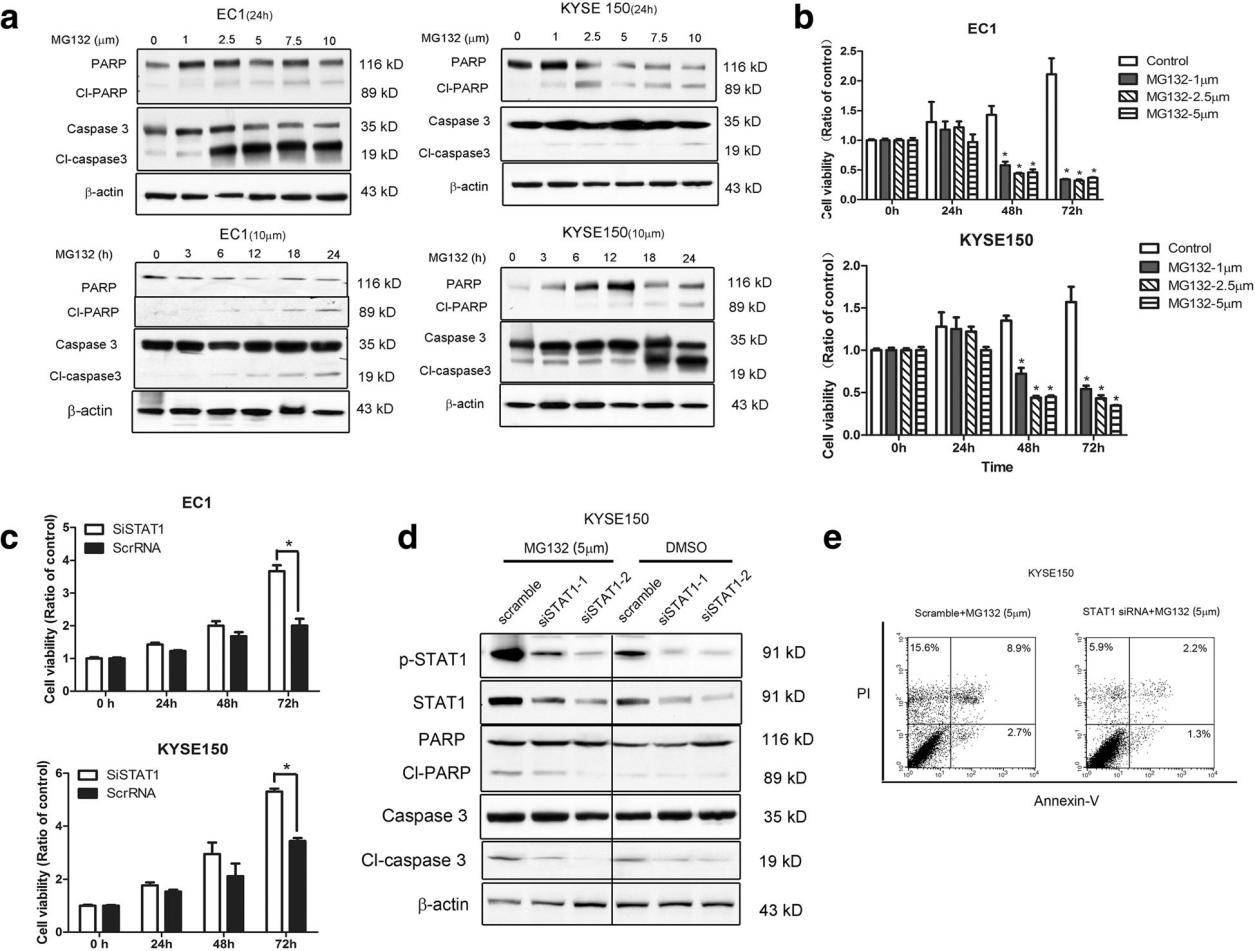
Silencing of STAT1 protein expression by siRNA attenuates the MG132-induced cell apoptosis. **a**. By Western blot analysis, we detected the expression of cleavage and total caspase 3 and PARP in EC1 and KYSE150 in a time- and dose-dependent MG132 treatment manner. **b.** Cell death induced by 1–5 μM MG132 was detected by MTS assay in both EC1 and KYSE150. **c**. MTS assay was used to detect the 5 μM MG132-induced cell death in both EC1 and KYSE150 cell lines with STAT1 siRNA and scrambled RNA (Scr RNA) transfection. **d**. KYSE150 cells were transfected with STAT1 siRNA or scrambled RNA and treated with 5 μM MG132 or control. The cleavage and total expression of PARP and caspase 3 was detected by using Western blot analysis. **e**. Apoptosis was detected by flow cytometry in KYSE150 cells transfected with STAT1 siRNA or scramble RNA. Values are expressed as means ± SD of three independent experiments. (*p < 0.05)

The observation that MG132 can induce effective apoptosis in various types of cancer, including ESCC, has been widely published [10, 14]. In view of our findings that STAT1 can induce effective apoptosis in ESCC [5], and our current observation that proteasome degradation is a key pathway to down-regulate STAT1, we hypothesized that MG132 also induces apoptosis in ESCC via a STAT1-dependent pathway. First, we confirmed that MG132 effectively induced apoptosis in ESCC cells, as evidenced by the reduction of viable cells as well as enhanced the cleavage of caspase 3 and PARP expression in dose and time dependent manner of MG132 treatment (Fig. 5a and b). Second, we found that siRNA knockdown of STAT1 significantly attenuated MG132-induced cell inhibition (at 5 μM) in EC1 and KYSE150 cells (Fig. 5c), and these findings correlated with the the lack of change in caspase 3, PARP and Annexin-V/propodium iodide (Fig. 5d-e). Taken together, these observations suggest that MG132-induced apoptosis in ESCC is highly STAT1-dependent.

### The expression and biological correlation between STAT1 and ERK in esophageal cancer

**Fig. 6:**
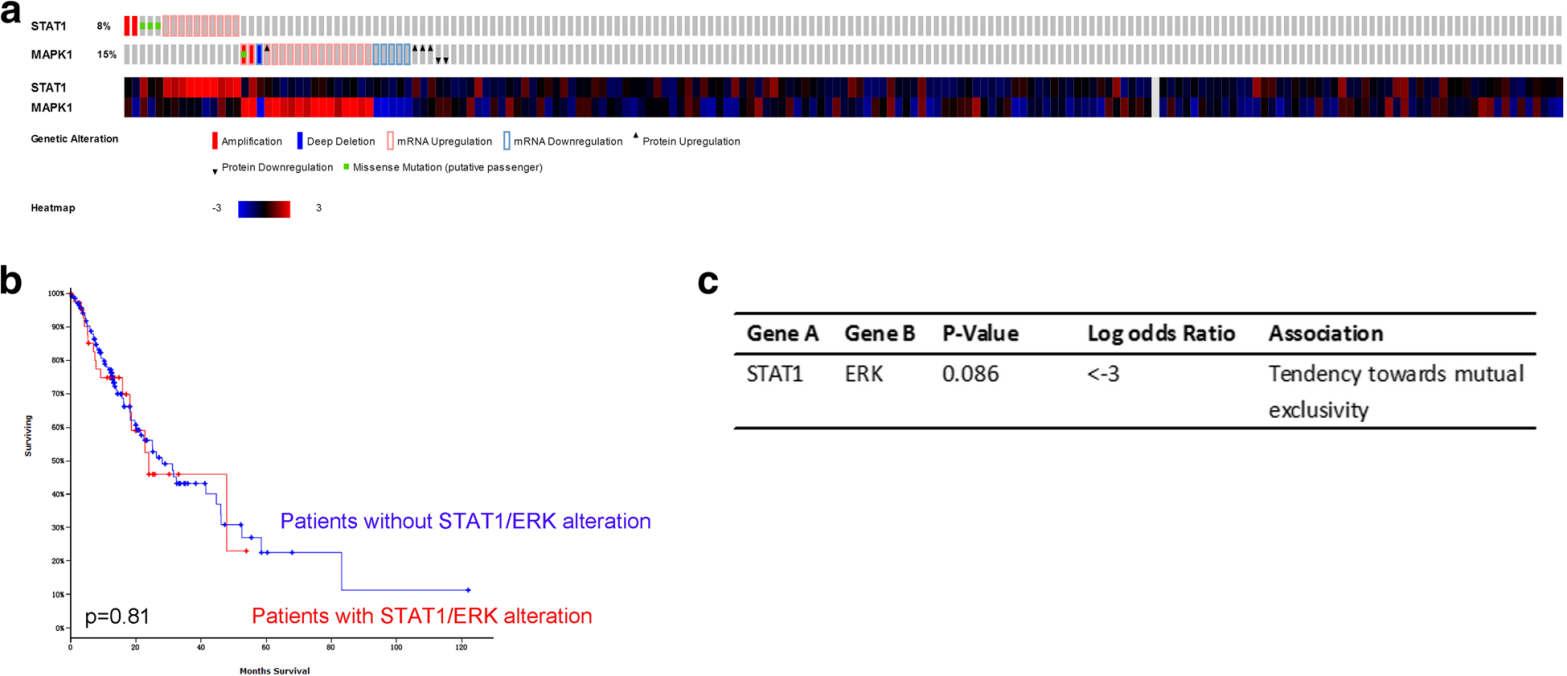
The expression and biological correlation of STAT1 and ERK in esophageal cancer. **a**. The expression of STAT1 and ERK(MAPK1) in 185 esophageal cancer samples from TCGA database. **b.** The survival data from these 185 esophageal cancer patients was shown when they were divided into patients with STAT1/ERK alteration and patients without. **c.** The mutual exclusivity and co-occurrence analysis from the CBioportal

In the previous published papers, we demonstrated the expression of STAT1 and ERK and its correlation with the clinical significance of ESCC patients [7]. Then we further investigate STAT1 and MAPK1(ERK) expression in ESCC. The bioinformatics analysis was performed to detect these gene alterations and expression in ESCC, using cBioportal Web resource online (cBioportal for Cancer Genomic)(Fig. 6a). However, we didn’t find the significance correlation between patients survival and STAT1/ERK alteration (Fig. 6b). As shown in Fig. 6c, the mutual exclusivity and co-occurrence analysis by Cbioportal revealed that STAT1 and ERK have a tendency to occur together. The results from the database supports our previous finding that ERK inhibit STAT1 expression and activation in ESCC and consistent with our previous results that ERK inhibited STAT1 expression [7].

## Discussion

In this paper, we have reported that the ERK negatively regulated IFN-γ signaling by ubiquitination of STAT1 in ESCC cells. We also found that MG132 induced apoptosis is partly dependent on STAT1activation, since STAT1 siRNA attenuate the apoptosis of ESCC cells.

The main objective of the current study is to identify the mechanism(s) that are responsible for the low expression of STAT1 in ESCC tumors. STAT1 has been reported as a tumor suppressor in multiple cancers by inhibiting tumor cell angiogenesis, tumorigenicity and metastasis [15]. One previous study has shown that reduced STAT1 expression in squamous cell carcinoma of the head and neck is due to gene methylation and silencing of the STAT1 gene [16]. Data from our experiments using 5-Aza does not support that STAT1 downregulation in ESCC was because of gene methylation. Based on another study with MEF cell lines in which ERK can promote STAT1 proteasomal, we also proved that ERK is responsible for the down-regulation of STAT1 in ESCC. In contrast with the model, ubiquitination and proteasomal degradation of STAT1 by ERK in ESCC cells are not dependent on STAT1 phosphorylation at Y701 and S727, two phosphorylation sites known to be functionally important for STAT1.

We also found evidence that mechanisms that constitutively activate STAT1 may exist in ESCC cells, since p-STAT1^Y701^ increased along with total STAT1 in all 3 ESCC cell lines examined. We believe that this finding carries important therapeutic implications. In view of the fact that STAT1 phosphorylation at tyrosine 701 is required for its dimerization, nuclear translocation and DNA-binding [17], a restoration of STAT1 expression in ESCC may be sufficient to induce apoptosis. The loss of STAT1 phosphorylation at serine 727 in EC1 but not the other 2 cell lines may have shed important insight into the biological heterogeneity of ESCC. Regarding p-STAT1^S727^, it is believed to boost the transcriptional activity of STAT1 and it has been implicated to enhance the anti-viral and anti-proliferation function of IFN-γ [18, 19]. While results from a study suggest that phosphorylation of STAT1^S727^ is dependent on IFN-γ-induced tyrosine phosphorylation of STAT1 [20], a few other studies have provided contradictory conclusions [21, 22]. This discrepancy may be linked to the observations that phosphorylation of STAT1^S727^ is mediated by different kinases stimulated by different signals [23–30]**.**

The ubiquitination and degradation of STAT1 has been described in a number of previous studies. Through our literature search, we found that the ubiquitin-proteasome pathway was implicated in the down-regulation of STAT1 activation by IFNγ [31]. It also has been reported that ubiquitination of STAT1 can be modulated in response to viral and parasitic infection [32, 33]. Several E3 ligases are known to mediate the ubiquitination and degradation of STAT1. For example, both STAT-interacting LIM (SLIM) and smad ubiquitination-regulating factor 1 (Smurf1) promote STAT1 ubiquitination and degradation in mouse macrophage cells and HEK293 cells [34–36]. In parallel with our observation, SLIM and Smurf1-mediated STAT1 degradation was found to be independent of STAT1 phosphorylation. Furthermore, in mouse embryonic fibroblasts, F-box E3 ligase and βTRCP were reported to promote STAT1 proteasomal degradation that is dependent on STAT1 phosphorylation at S727 [11]**.**

In addition to promoting the proteasomal degradation of STAT1, our data suggested that ERK dampens STAT1 activation by suppressing the production of IFNγ, which is known to be a potent activator of STAT1. IFNγ is used for treating cancers and viral infection, however, the therapeutic efficacy of IFNγ is highly variable among different types of cancer [37, 38]**.** For example, while IFNγ appears to be effective in treating adult T cell leukemia and ovarian cancers, it is relatively ineffective for most patients suffering from chronic myeloid leukemia [37]. Moreover, IFNγ-treated melanoma patients were found to have a worse survival than those who did not receive IFNγ [38]. Based on our result, IFNγ might be an effective anti-cancer drug for tumors that lack a high level of expression and/or activation of ERK, and/or express a certain basal level of STAT1 that can be activated.

## Conclusion

In this study, we demonstrated that ERK promotes proteasome degradation of STAT1 and down-regulates STAT1 activation by suppressing IFNγ production. We previous found that STAT1 plays a tumor suppressor role in esophageal cancer, and ERK expression was inversely correlated with STAT1. In this study, we further found that ERK promotes tumorigenesis of ESCC by suppressing the expression and activation of STAT1.

## Additional file

Additional file 1: **Figure S1.** The expression of STAT1 in ESCC cell lines has no change via 5-Aza treatments. **A**. EC1, KYSE150 cell lines were treated with 10 μM of 5-Aza for 24 h, RNA level of STAT1 was analyzed by qRT-PCR. **B**. EC1, KYSE150 cell lines were treated with 10 μM of 5-Aza for 24 h, Western blot analysis of p-STAT1 and STAT1 in total cell lysates were shown. (JPG 695 kb)

## Abbreviations

5-Aza: 5-Aza-2′-deoxycytidine
DMEM: Dulbecco’s modified Eagle’s medium
ELISA: Enzyme-linked immunosorbent assay
ERK: Extracellular regulated protein kinases
ESCC: Esophageal squamous cell carcinoma
IFNγ: gamma-interferon
IP: Immunoprecipitation
MAPK: Mitogen Activated Protein Kinase
MEF: Mouse embryonic fibroblast
MEK1: Mitogen-activated protein kinase 1
MG132: N-carbobenzoxyl-L-leucinyl-L-norleucinal
MTS: 3-(4,5-dimethylthiazol-2-yl)-5-(3-carboxymethoxyphenyl)-2-(4-sulfophenyl)-2H-tetrazolium
STAT: Signal transducers and activators of transcription
UPP: Ubiquitin-proteasome pathway
