# Supplementary figures and images for: ERK is a negative feedback regulator for IFN-γ/STAT1 signaling by promoting STAT1 ubiquitination
Source: BMC Cancer. 2018 May 31;18:613. doi: 10.1186/s12885-018-4539-7 (PMC5984314; doi:10.1186/s12885-018-4539-7)

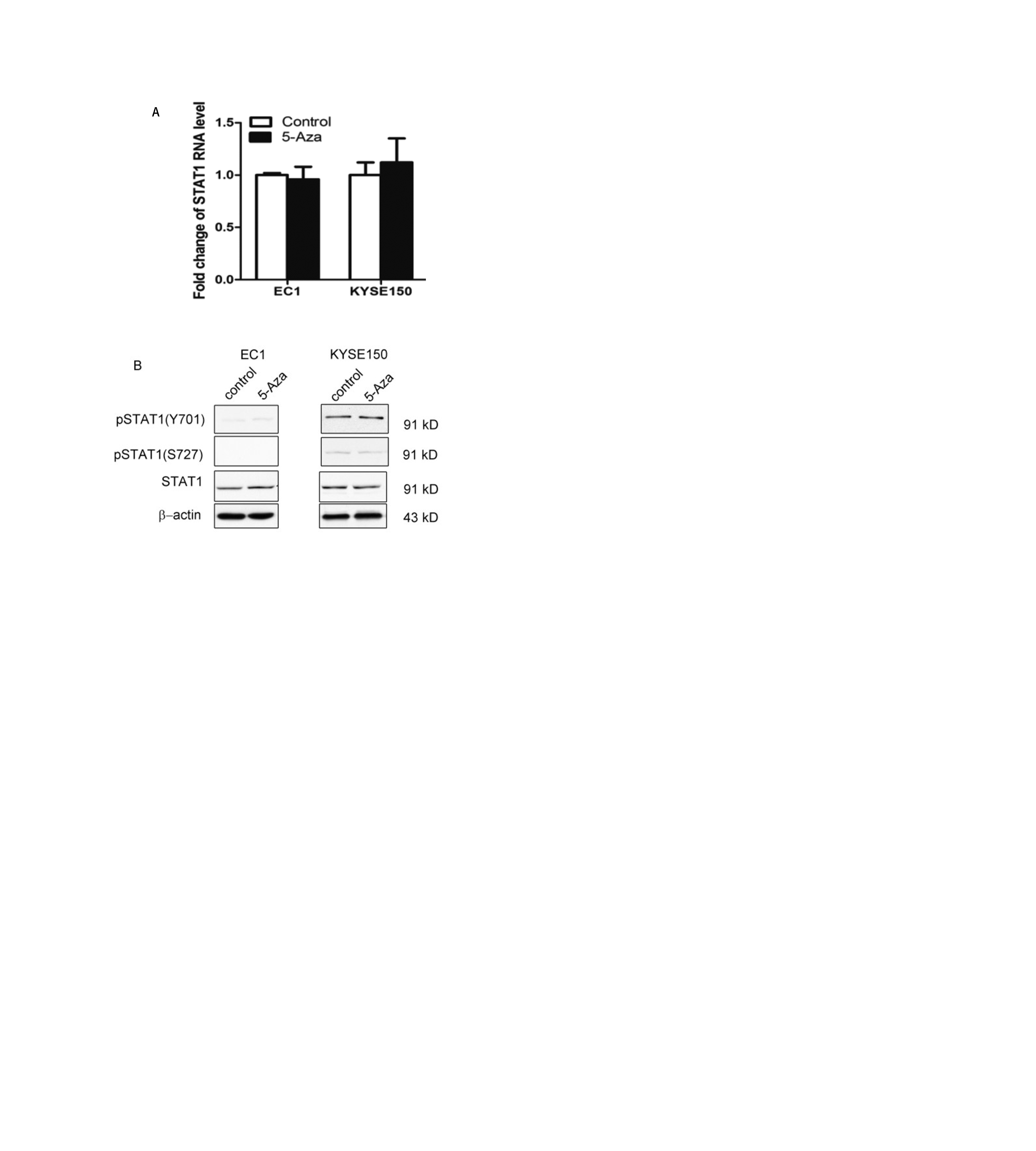

Supplement: Supplementary file 1 — Figure S1. The expression of STAT1 in ESCC cell lines has no change via 5-Aza treatments. A. EC1, KYSE150 cell lines were treated with 10 μM of 5-Aza for 24 h, RNA level of STAT1 was analyzed by qRT-PCR. B. EC1, KYSE150 cell lines were treated with 10 μM of 5-Aza for 24 h, Western blot analysis of p-STAT1 and STAT1 in total cell lysates were shown. (JPG 695 kb) [file 12885_2018_4539_MOESM1_ESM.jpg]
